# Metformin attenuates blood-brain barrier disruption in mice following middle cerebral artery occlusion

**DOI:** 10.1186/s12974-014-0177-4

**Published:** 2014-10-15

**Authors:** Yanqun Liu, Guanghui Tang, Yaning Li, Yang Wang, Xiaoyan Chen, Xiang Gu, Zhijun Zhang, Yongting Wang, Guo-Yuan Yang

**Affiliations:** Department of Neurology, Ruijin Hospital, School of Medicine, Shanghai Jiao Tong University, Shanghai, 200025 China; Neuroscience and Neuroengineering Research Center, Med-X Research Institute and School of Biomedical Engineering, Shanghai Jiao Tong University, Shanghai, 200030 China; Department of Neurology, Med-X Research Institute and School of BME, Ruijin Hospital, School of Medicine, Shanghai Jiao Tong University, 1954 Hua-shan Road, Shanghai, 200030 China

**Keywords:** Blood-brain barrier, ICAM-1, Inflammation, Ischemic stroke, Metformin

## Abstract

**Background:**

Metformin, a widely used hypoglycemic drug, reduces stroke incidence and alleviates chronic inflammation in clinical trials. However, the effect of metformin in ischemic stroke is unclear. Here, we investigated the effect of metformin on ischemic stroke in mice and further explored the possible underlying mechanisms.

**Methods:**

Ninety-eight adult male CD-1 mice underwent 90-minute transient middle cerebral artery occlusion (tMCAO). Metformin (200 mg/kg) was administrated for up to 14 days. Neurobehavioral outcomes, brain infarct volume, inflammatory factors, blood-brain barrier (BBB) permeability and AMPK signaling pathways were evaluated following tMCAO. Oxygen glucose deprivation was performed on bEND.3 cells to explore the mechanisms of metformin in inhibiting inflammatory signaling pathways.

**Results:**

Infarct volume was reduced in metformin-treated mice compared to the control group following tMCAO (*P* < 0.05). Neurobehavioral outcomes were greatly improved in metformin-treated mice (*P* < 0.05). MPO^+^ cells, Gr1^+^ cells, MPO activity and BBB permeability were decreased after metformin administration (*P* < 0.05). In addition, metformin activated AMPK phosphorylation, inhibited NF-κB activation, down-regulated cytokine (IL-1β, IL-6, TNF-α) and ICAM-1 expression following tMCAO (*P* < 0.05). Furthermore, metformin activated AMPK signaling pathway and alleviated oxygen-glucose deprivation-induced ICAM-1 expression in bEND.3 cells (*P* < 0.05). Compound C, a selective AMPK inhibitor, eliminated this promotional effect.

**Conclusions:**

Metformin down-regulated ICAM-1 in an AMPK-dependent manner, which could effectively prevent ischemia-induced brain injury by alleviating neutrophil infiltration, suggesting that metformin is a promising therapeutic agent in stroke therapy.

**Electronic supplementary material:**

The online version of this article (doi:10.1186/s12974-014-0177-4) contains supplementary material, which is available to authorized users.

## Background

Ischemic stroke is the second leading cause of death worldwide [1]. Due to its high disability, it is also a big burden on our society. So far, the only Food and Drugs Administration (FDA) approved drug for the treatment of ischemic stroke is rtPA, which improves clinical outcomes if administrated within 4.5 hours after the stroke onset. However, due to its narrow therapeutic window, less than 5% of patients benefit from it [2]. Therefore, developing effective drugs to treat ischemic stroke is an important task.

Metformin is a drug widely prescribed for the treatment of type 2 diabetes and other metabolic syndromes since 1960s [3]. Through activating AMP-activated kinase (AMPK), metformin inhibits hepatic glucose production and increases peripheral glucose utilization, which effectively controls blood glucose level [4]. However, its ability is not limited to lowering glucose. The benefits of metformin have been demonstrated in clinical trials. Metformin reduces stroke incidence and diabetes related death [5]. Metformin also reduces intercellular adhesion molecule-1 (ICAM-1) and vascular cell adhesion molecule-1 (VCAM-1) levels in plasma and alleviates chronic inflammation in patients [6]. Nevertheless, these effects were independent of its glycemic management properties, suggesting that metformin may have other functions through mechanisms other than glucose reduction. AMPK is a trimetric enzyme comprising a catalytic α-subunit and regulatory β- and γ-subunits [7]. An alteration in AMP/ATP ratio activates AMPK and promotes AMPK phosphorylation at a threonine residue (Thr-172) [3]. A series of pathological conditions such as glucose deprivation, ischemia, starvation and oxidative stress increase AMPK activity [8]. Agents such as resveratrol and adiponectin can also activate AMPK. AMPK activation is a protective reaction that occurs after injury. AMPK activated by metformin showed to reduce endothelial cell apoptosis and diminish cardiomyocyte death [9,10]. It has been demonstrated that increasing AMPK activity in neurons protects neurons from various injuries [11]. In addition, metformin reduced TNF-α-induced inflammation via activation of AMPK in vascular endothelial cells (ECs) [12]. It has been indicated that AMPK activation in cells in the immune system promotes the switch from a pro-inflammatory to an anti-inflammatory phenotype [13]. Thus, metformin could be a promising anti-inflammatory agent.

ICAM-1 is expressed on many cell types including ECs and lymphocytes [14]. ICAM-1 is expressed constitutively on ECs at low level and its expression is significantly increased in hypoxic condition [15]. ICAM-1 expressed on ECs facilitates neutrophil adhesion and tissue infiltration, which play critical roles in the progress of ischemic stroke [16,17]. Infiltrated leukocytes induce a secondary injury after reperfusion by producing detrimental substances that damage brain cells and disrupt the blood-brain barrier (BBB) [18,19]. BBB disruption after ischemia increases brain edema and exacerbates ischemic injury [14]. Since ICAM-1 plays a vital role in neutrophil infiltration and cerebral injury after reperfusion, it is a promising target in the treatment of ischemic stroke.

Studies have illustrated that metformin provides cardioprotection against myocardial infarction [4]. However, the function of metformin in inflammation after ischemic stroke is unknown. In our research, we explored whether metformin could reduce ischemic brain injury using a mouse transient middle cerebral artery occlusion (tMCAO) model, and attempted to define the underlying mechanism of metformin.

## Methods

### Experimental design

**Figure 1:**
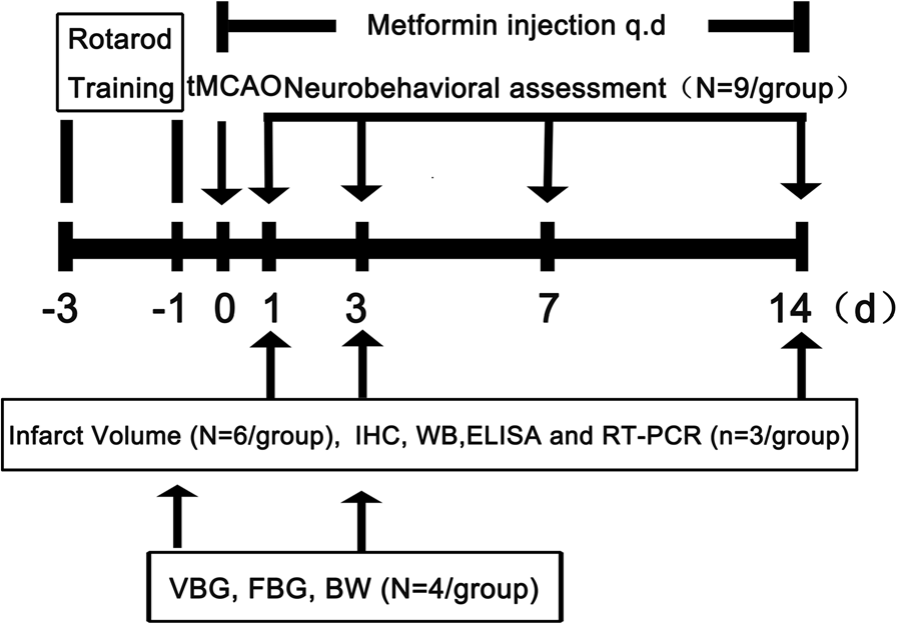
**Experimental design.** Graph illustrating the experimental design including transient middle cerebral artery occlusion (tMCAO), metformin administration, infarct volume, protein expression and neurobehavioral assessments.

Animal protocol was approved by the Institutional Animal Care and Use Committee of Shanghai Jiao Tong University, Shanghai, China. Ninety-eight adult male CD1 mice were divided into two groups that either underwent metformin or saline treatment. At 1 and 3 days after tMCAO, mice were sacrificed and samples were collected for further study. Metformin (Sigma, St. Louis, MO, USA) was dissolved in sterile saline at a concentration of 30 mg/ml and 200 mg/kg was administered intra-peritoneally immediately after reperfusion and then administered daily until the animals were sacrificed. An equal volume of saline was used for the control group. The dose was chosen according to a previous study [20]. The whole experimental design and the number of animal used are displayed in Figure 1.

### Transient middle cerebral artery occlusion (tMCAO) in mice

tMCAO was carried out as previously described [21]. Adult CD1 mice weighing 30 ± 5 grams were anesthetized with ketamine/xylazine (100 mg/10 mg/kg; Sigma, St. Louis, MO, USA) through intra-peritoneal injection. After isolation of the left common carotid artery, the external carotid artery (ECA) and the internal carotid artery (ICA), a silicone-coated 6-0 suture (Covidien, Mansfield, MA, USA) was gently inserted into the ICA and stopped at the opening of the middle cerebral artery (MCA). Successful occlusion was ascertained by a decrease of surface cerebral blood flow to 10% of baseline using a laser Doppler flowmetry (Moor Instruments, Devon, UK). Reperfusion was performed 90 minutes after tMCAO with suture withdrawal. Sham-operated mice underwent the same procedure except for the insertion of the suture into the ICA. The mortality in our study was less than 5%.

### Infarct volume measurement

Infarct volume was measured using cresyl violet (Sigma, St. Louis, MO, USA) staining as previously described [22]. The ischemic area of each section was depicted by image analysis software (Image J, NIH, MD, USA). Infarct volume was calculated as described in our previous study [23].

### Neurobehavioral assessments

Neurobehavioral assessments were conducted by an experimenter who was blind to the treatment conditions. The rotarod test was used to evaluate the motor and balance functions of the mice. The mice were trained to stay on an accelerating rotating cylinder for 3 days before tMCAO, and time remained on the rotating rod was recorded before surgery and at 1, 3, 7 and 14 days after surgery. The velocity was increased slowly from 4 to 40 rpm within 2 minutes. For each test, every animal was tested three times, and the average time maintained on the rod was recorded. For neurological function assessment, a modified Neurological Severity Scores (mNSS) ranging from 0 to 14 score was adopted, which included raising the mouse by the tail (0 to 3), walking on the floor (0 to 3), beam balance tests (0 to 6), and the relaxes absence (0 to 2) [22].

### Immunostaining

Double staining: ZO-1/CD31, occludin/CD31 or claudin-5/CD31 double staining was conducted as previously described [23]. Briefly, brain sections were blocked with 10% FBS for 1 hour and then incubated with ZO-1 (1:100 dilution, Invitrogen, Carlsbad, CA, USA) and CD31 (1:200 dilution, R&D Systems, Minneapolis, MN, USA); occludin (1:100 dilution, Invitrogen, Carlsbad, CA, USA) and CD31; claudin-5 (1:100 dilution, Invitrogen, Carlsbad, CA, USA) and CD31 at 4°C overnight. After washing, brain sections were incubated with the appropriate second antibodies for 1 hour. Brain sections were examined using a confocal microscope (Leica, Solms, Germany) and photographs were taken for further analysis.

DAB staining: for myeloperoxidase (MPO), ICAM-1 and Gr1 (Ly 6G) immunostaining, brain sections were incubated in 0.3% H_2_O_2_ in methanol for 30 minutes. After blocking with FBS, the primary anti-body MPO (1:300 dilution, R&D Systems, Minneapolis, MN, USA) and ICAM-1 (1:200 dilution, R&D Systems, Minneapolis, MN, USA), Gr1 (1:100 dilution, Millipore, Darmstadt, Germany) were incubated overnight at 4°C. Sections were incubated with biotinylated-conjugated secondary antibody (Vector Laboratories, Burlingame, CA, USA) and then incubated with Vectastain ABC Reagent. The reaction product was visualized using a DAB substrate (Vector Laboratories, Burlingame, CA, USA). Eight interested fields in each ipsilateral hemisphere, including the perifocal region in both cortex and striatum, were photographed in each section and five consecutive sections spaced at 200 μm were counted in each mouse. MPO^+^ and Gr1^+^ (Ly 6G) cells were counted in each field by a person blinded to the treatment group. IgG leakage was examined as previously reported and the procedure was similar to MPO staining except for the primary antibody incubating process [23]. Four areas of ischemic penumbra from each slide were photographed. And mean optical density was measured using Image-Pro Plus software (Media Cybernetics, Bethesda, MD, USA).

### Western blot analysis

Samples were lysed in radioimmunoprecipitation assay (RIPA) (Millipore, Bedford, MA, USA) supplemented with 1 mmol/L PMSF (Thermo, Waltham, MA, USA), cocktail (Thermo, Waltham, MA, USA) and phosphatase inhibitor (Thermo, Waltham, MA, USA). For Western blot analyses, samples containing the same amount of proteins were loaded onto the resolving gel (Promoton, Shanghai, China) for electrophoresis after denaturing. Proteins were transferred onto a nitrocellulose membrane (Whatman, Piscataway, NJ, USA). After being blocked with 5% non-fat milk, the membrane was incubated with primary antibodies at the following dilution MPO (1:500), ICAM-1 (1:2,000), ZO-1 (1:500), occludin (1:500), claudin-5 (1:500), p-AMPK (1:1,000, Cell Signaling Technology, Beverly, MA, USA), AMPK (1:1,000, Cell Signaling Technology, Beverly, MA, USA), p-NF-κB (1:1,000, Cell Signaling Technology, Beverly, MA, USA), NF-κB (1:1,000, Cell Signaling Technology, Beverly, MA, USA), β-actin (1:1,000, Santa Cruz Technology, Santa Cruz, CA, USA) at 4°C overnight, respectively. After washing, the membrane was incubated with the appropriate horseradish peroxidase (HRP)-conjugated secondary antibody for 1 hour and then reacted with enhanced chemiluminescence substrate (Pierce, Rockford, IL, USA). The results were recorded by Quantity One image software (Bio-Rad, Hercules, CA, USA) and relative intensity was calculated using Gel-Pro Analyzer software (Media Cybernetics, Bethesda, MD, USA).

### MPO activity assay

MPO activity assay was performed as described previously [23]. In brief, brain protein (10 μL) from ipsilateral hemisphere was added to 180 μL of work solution, which contained 2 mmol/L O-dianisidin-dihydrochloride (Sigma) dissolved in 180 μl of 50 mmol/L potassium phosphate buffer (pH =6). Before measurement, 10 μL of 100 mmol/L H_2_O_2_ was added. Changes in absorbance at 460 nm over 10 minutes were measured. MPO activity was expressed as U/mg tissue, and 1U of MPO activity represents the amount of enzyme degrading 1 μmol H_2_O_2_ per minute at 25°C.

### Real-Time PCR

Total RNA from the ischemic hemisphere was extracted using TRIzol reagent (Invitrogen, Carlsbad, CA, USA) and dissolved in 60 μL RNA free water according to the manufacturer’s instructions. A universal 2-step RT-PCR cycling condition was used: 95°C for 30 seconds followed by 40 cycles of 95°C for 5 seconds and 60°C for 30 seconds. mRNA levels were normalized to the endogenous control, GAPDH expression, and were calculated using fold change relative to the saline control group [23].

### Enzyme Linked Immunosorbent Assay (ELISA) analysis

Protein levels of IL-1β, IL-6, and TNF-α were quantified using an ELISA kit (R&D systems, Minneapolis, MN, USA) according to the manufacturer’s instruction. Absorbance at 450 nm was recorded and the concentration of the target protein was read according to the standard curve. Result was expressed as pg/mg protein.

### Evans blue extravasation

Evans blue extravasation was measured as previously described. In brief, 3 days after tMCAO, 4 ml/kg of 2% Evans blue (Sigma, St. Louis, MO, USA) in saline was administered intraperitoneally. After 2 hours circulation, mice were anesthetized and perfused with saline through the left ventricle until colorless fluid outflowed from the right atrium. Then, ipsilateral and contralateral hemispheres were collected after decapitated. Each hemisphere was weighed rapidly, homogenized in 1 ml of 50% trichloroacetic acid (wt/vol). After centrifugation (12,000 × *g*, 20 minutes), supernatant was collected and mixed with ethanol (1:3). The concentration of Evans blue was determined by measuring the 610 nm absorbance and tissue content of Evans blue was quantified from a linear standard curve and expressed in terms of Evans blue (μg)/tissue (g).

### Oxygen glucose deprivation

bEND.3 was purchased from American Type Culture Collection (ATCC) and cultured in DMEM (Gibco Laboratories, Grand Island, NY, USA) supplemented with 10% FBS. Ischemia-like conditions *in vitro* were induced by oxygen glucose deprivation and reperfusion-like conditions *in vitro* were induced by reoxygenation. After cells reached a 90% confluence, the medium was replaced with DMEM without glucose. Then, cultures were transferred to an anaerobic chamber infused with a gas mixture containing 5% CO_2_, 95% N_2_. After incubating for 6 hours, cells were further cultured in DMEM supplemented with 10% FBS under normal conditions for another 24 hours with or without metformin. p-AMPK analysis in bEND.3 cells was determined 1 hour, 4 hours, and 24 hours after 6 hours oxygen glucose deprivation (OGD). The dose used was 10 mM, which was chosen according to previous reports [20]. Compound C (Sigma, St. Louis, MO, USA), a selective AMPK inhibitor was added to the medium at a final concentration of 10 μmol/L before OGD treatment and maintained throughout the whole experiment [24]. An equal volume of PBS was used in the control group.

### Statistical analysis

Results were presented as mean ± SD. Statistical analysis was evaluated by Prism 4 software (GraphPad Software, San Diego, CA, USA). For comparison between the two groups, statistical significance was determined through a Student's t test. For comparison among multiple groups, statistical significance was evaluated using one-way ANOVA followed by a Student-Newman-Keuls test. A probability value of *P* < 0.05 was considered statistically significant.

## Results

### Metformin reduced infarct volume and improved neurobehavioral outcomes

**Figure 2:**
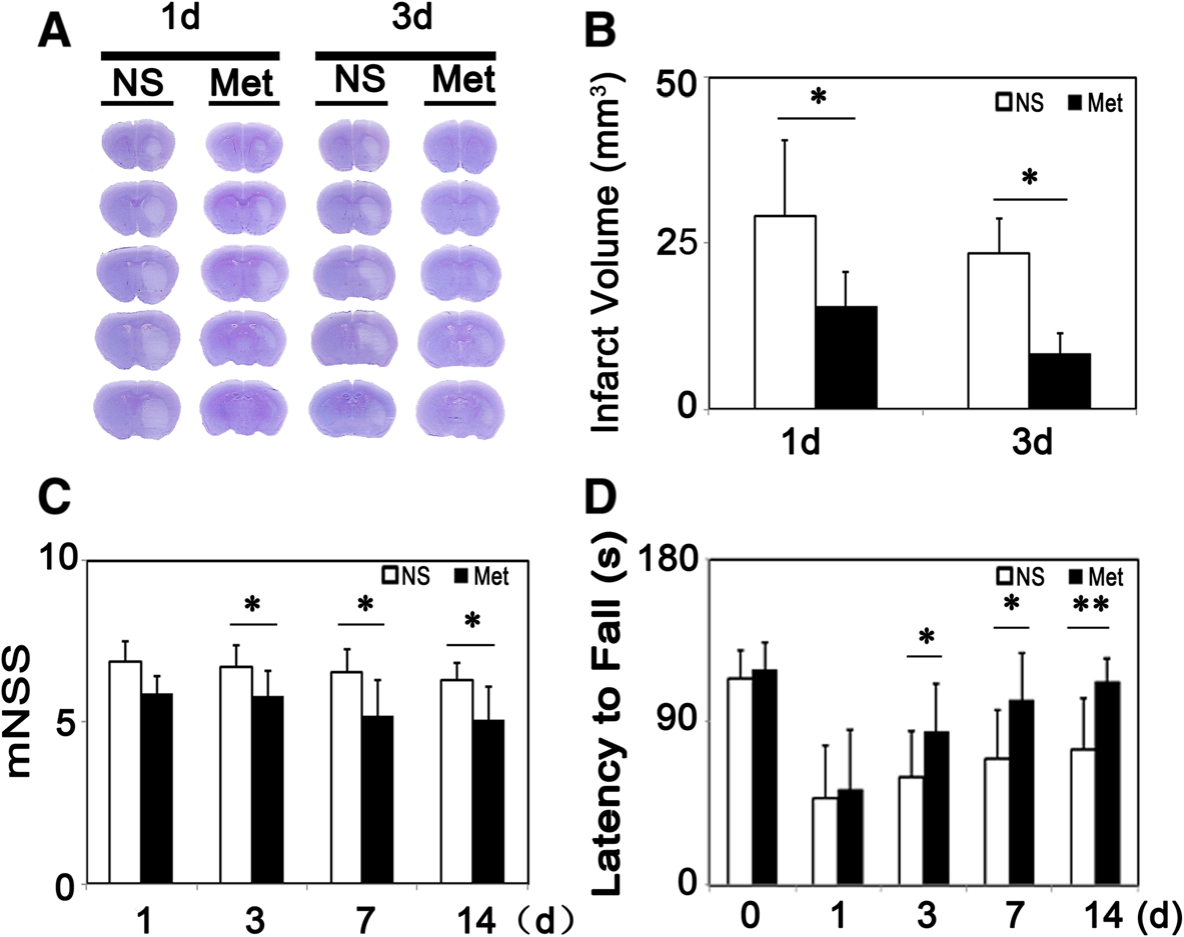
**Metformin reduced infarct volume and improved neurobehavioral outcomes following transient middle cerebral artery (tMCAO) in mice. (A)** Photographs show a series of coronal sections with cresyl violet staining following tMCAO in metformin- and saline-treated mice. **(B)** Bar graph shows a quantification of the infarct volume from (A). n =6 per group. The changes in neurological scores **(C)** and the rotarod test **(D)** at 1, 3, 7 and 14 days following tMCAO in metformin- and saline-treated mice. n =9 per group, data are mean ± SD, *P < 0.05, metformin versus control group, **P < 0.01, metformin versus control group.

We found that after metformin treatment, infarct volume was reduced at 1 and 3 days after tMCAO (Figure 2A-B). To further explore the function of metformin, we used mNSS to examine the motor, balance and reflex functions of mice after tMCAO. We showed that mice had significantly lower scores after 3 days in the metformin-treated group after tMCAO for at least 14 days (Figure 2C). A similar result was obtained from the rotarod test (Figure 2D). To investigate whether metformin influenced glucose levels after tMCAO, fasting blood-glucose (FBG) was tested before, 3 days and 14 days after tMCAO, indicating metformin did not influence FBG level, blood gas or body weight after both 3 (Table 1) days and 14 days treatment (Data were shown in Additional file 1: Table S1)

**Table 1:** **Metformin did not influence blood gas, glucose level and body weight in transient middle cerebral artery occlusion (tMCAO) mice**

	**Before tMCAO**		**After tMCAO**	
	**NS**	**Met**	**NS**	**Met**
pH	7.35 ± 0.05	7.34 ± 0.03	7.34 ± 0.07	7.35 ± 0.03
PCO_2_ (mmHg)	38 ± 5	33 ± 3	30 ± 7	30 ± 5
PO_2_ (mmHg)	62 ± 4	60 ± 7	64 ± 2	66 ± 12
SO_2_ (%)	91 ± 8%	87 ± 10%	91 ± 2%	91 ± 5%
Na(mmol/L)	155 ± 2	153 ± 2	154 ± 3	152 ± 2
K (mmol/L)	3.2 ± 0.1	3.1 ± 0.4	3.5 ± 0.3	3.9 ± 0.2
iCa (mmol/L)	1.37 ± 0.02	1.34 ± 0.06	1.22 ± 0.04	1.26 ± 0.04
Glu (mg/dL)	132 ± 20	127 ± 33	123 ± 16	129 ± 24
Hct (%PCV)	37 ± 1%	35 ± 1%	34 ± 6%	35 ± 2%
Hb (g/dL)	12.4 ± 0.4	11.9 ± 0.3	11.4 ± 2.2	11.8 ± 0.7
Body weight (g)	33 ± 2	32 ± 1	25 ± 6	28 ± 4

Table showed vein blood gas analysis results, glucose levels and body weight at 1 day before tMCAO and at 3 days after tMCAO in metformin-(Met) and saline(NS)-treated mice (n =4 per group). Data were mean ± SD.

Legend: Glu, glucose; Hb, hemoglobin; Hct, hematocrit; PCO_2_, partial pressure of CO_2_; PO_2_, partial pressure of O_2_; PCV, packed cell volume; SO_2_, oxygen saturation.

### Metformin alleviated neutrophil infiltration and IL-1β, IL-6, TNF-α expression

**Figure 3:**
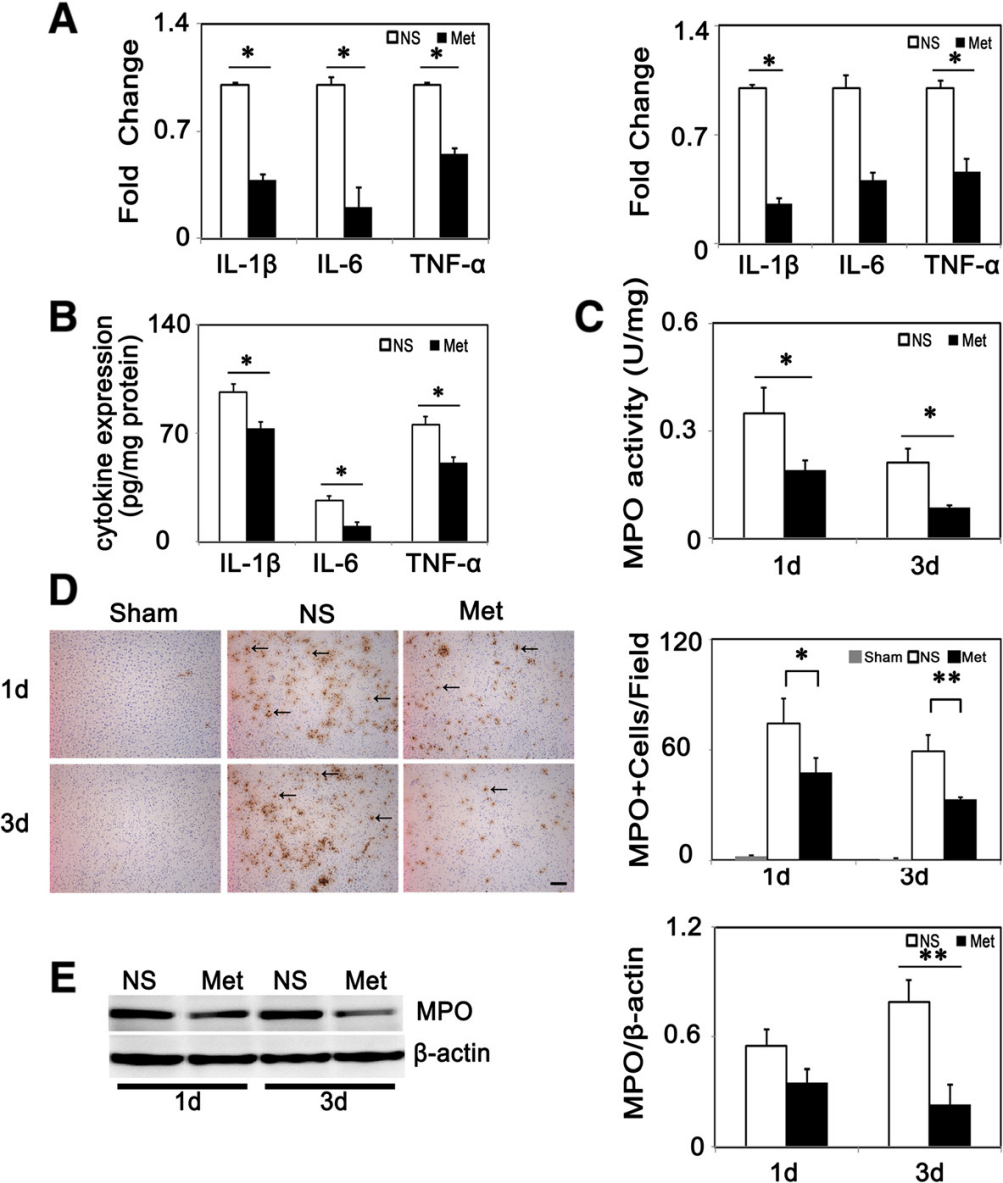
**Metformin alleviated neutrophil infiltration and inflammatory cytokine expression in mice. (A)** Relative fold changes of inflammatory cytokines (IL-1β, IL-6, TNF-α in metformin and control mice at 1 (left) and 3 days (right) following tMCAO (n =3 per group). **(B)** Protein level of inflammatory cytokines IL-1β, IL-6, TNF-α expression in metformin and control mice at 3 days following tMCAO (n =3 per group). **(C)** Bar graph shows MPO activity in metformin and control mice (n =3 per group). **(D)** MPO^+^ cells (arrows) and their quantification in sham group, control and metformin-treated mice at 1 and 3 days following tMCAO. Scale bar =100 μm. **(E)** Western blot of MPO expression in metformin and control mice. Bar graph shows a quantification of MPO. Data are mean ± SD, *P < 0.05, **P < 0.01, metformin versus control group.

To investigate the effect of metformin on neutrophil infiltration in the acute phase of cerebral ischemia, we performed 3,3’-diaminobenzidine (DAB) staining to detect MPO^+^ cells. Results showed MPO^+^ cells were almost undetectable in the sham group, and there was a decrease in MPO^+^ cells at 1 and 3 days after tMCAO in the metformin-treated group compared to the control group (Figure 3D). Western blot analysis indicated that MPO were reduced in metformin-treated mice (*P* < 0.01, Figure 3E). MPO activity is an indicator of inflammation and could be used to evaluate neutrophil accumulation [23]. MPO activity was attenuated in the metformin-treated group compared to the control group (*P* < 0.05, Figure 3C). In addition, we used another neutrophil marker Gr1 (Ly 6G) to evaluate neutrophil infiltration after cerebral ischemia. Results showed that metformin reduced Gr1 positive cells effectively at 1 day and 3 days after tMCAO (Additional file 2: Figure S1, *P* < 0.01). We further used RT-PCR and ELISA to evaluate changes in inflammation-related cytokine expression in mRNA and protein levels. Metformin reduced IL-β, IL-6, TNF-α mRNA at 1 day after tMCAO. Although there was a downward tendency in IL-6 mRNA, only changes in IL-β, and TNF-α were statistically significant in the metformin group compared to the control group at 3 days after tMCAO (Figure 3A). There was a decrease of IL-β, IL-6, TNF-α expression at 3 days after tMCAO in protein level (Figure 3B, *P* < 0.01).

### Metformin reduced BBB disruption

**Figure 4:**
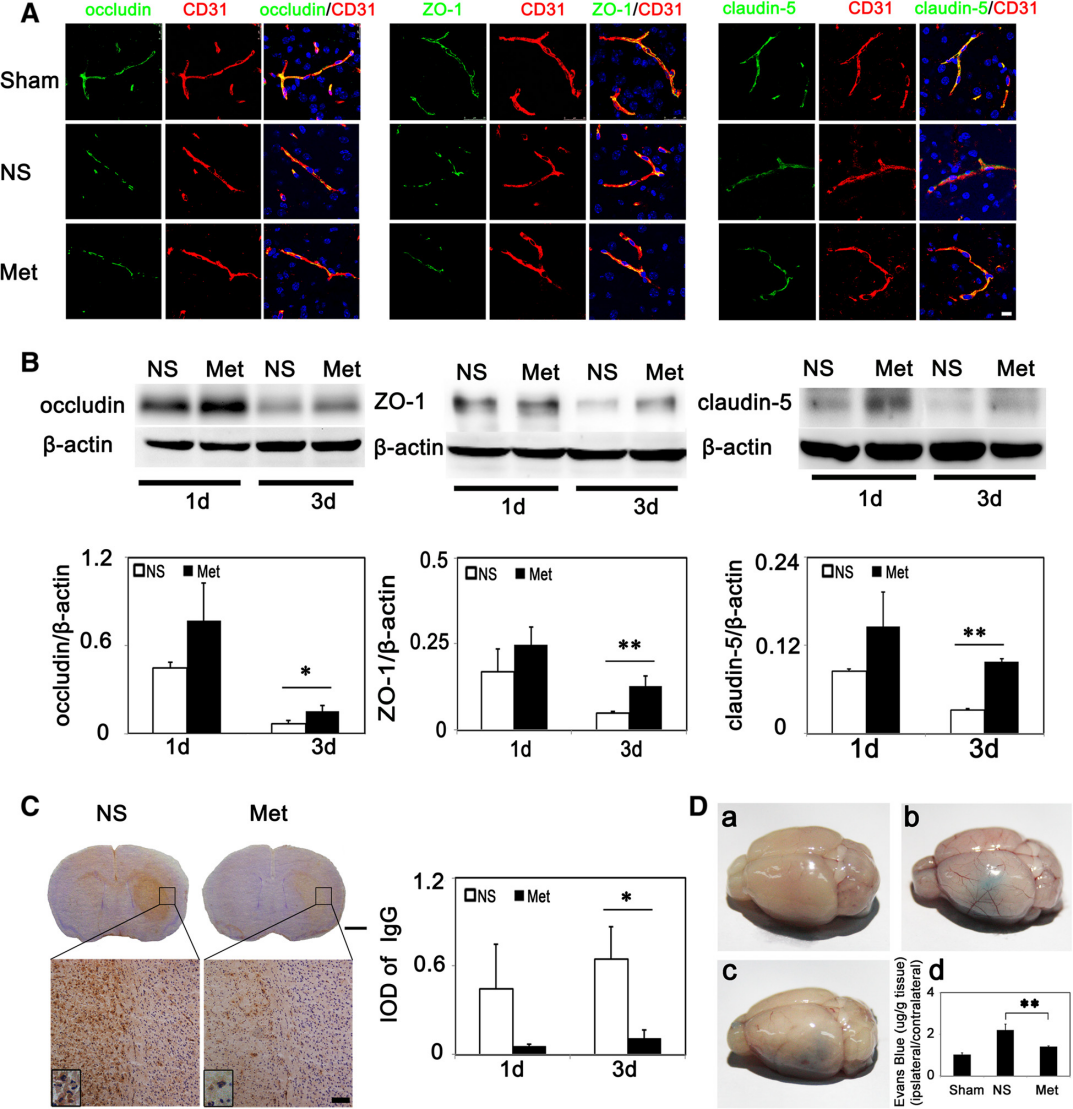
**Metformin promoted ZO-1, occludin and claudin-5 rearrangement and lessened IgG and Evans blue extravasation. (A)** Occludin, ZO-1 and claudin-5 expression in sham group, NS and metformin-treated mice at 3 days following tMCAO. Scale bar =10 μm. **(B)** Representative result of occludin, ZO-1 and claudin-5 expression at 1 and 3 days after tMCAO. Bar graphs show a quantification of occludin, ZO-1 and claudin-5 expression. **(C)** IgG leakage at 3 days following tMCAO in saline and metformin-treated group. Higher magnifications are shown below. Boxes displayed representative IgG staining. Scale bar =1 mm (upper) and 100 μm (lower). Bar graph shows a semi-quantification of integrated optical density (IOD) of IgG at 1 and 3 days after tMCAO (n =3 per group). **(D)** Images show Evans blue extravasation in sham (a), saline (b) and metformin (c) group at 3 days after tMCAO. Blue area indicates extravasation of Evans blue. Bar graph shows a quantification analysis of Evans blue contents in brain tissue (n =3 per group). Data were mean ± SD, *P < 0.05, metformin versus saline group, **P < 0.01, metformin versus saline group.

To evaluate endothelial cell permeability after metformin treatment, we conducted occludin/CD31, ZO-1/CD31 and claudin-5/CD31 double staining to observe tight junction distribution *in situ* at 3 days after tMCAO. Result indicated that occludin and ZO-1 were continuously located on the margin of ECs in sham group, claudin-5 was continuously located along ECs, and fewer gaps were formed in the metformin-treated group (Figure 4A). Gap formation and rearrangement were used to evaluate tight junction disruption after injury. To evaluate tight junction rearrangement, Western blot was adopted and we found that metformin-treated mice demonstrated occludin, ZO-1 and claudin-5 hyper-expression (Figure 4B). In addition, we performed IgG immunostaining and Evans blue extravasation to evaluate endothelial permeability and found that there was significantly reduced IgG and Evans blue leakage at 3 days after tMCAO in metformin-treated mice (Figure 4C-D).

### Metformin down-regulated ICAM-1 expression via AMPK signaling pathway

**Figure 5:**
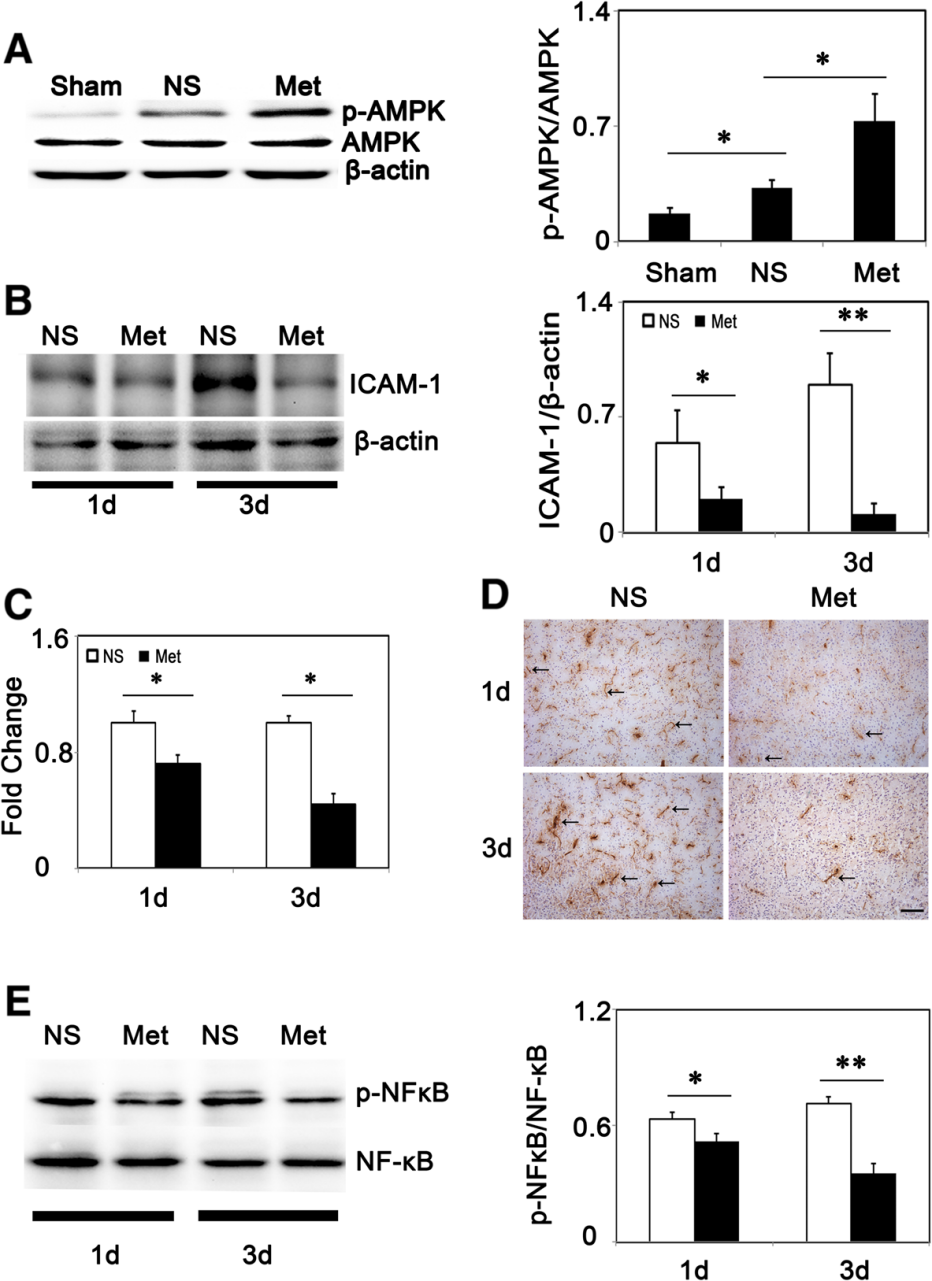
**Metformin promoted phosphorylation of AMPK and reduced ICAM-1 expression in transient middle cerebralartery occlusion (tMCAO) mice. (A)** p-AMPK and AMPK expression in sham, saline- and metformin-treated groups at 1 day after tMCAO. Bar graph showed a quantification of p-AMPK/AMPK ratio (n =3/group). **(B)** ICAM-1 expression and quantification at 1 and 3 days after tMCAO in metformin and control mice (n =3 per group). **(C)** Fold change of ICAM-1 expression in mRNA level in metformin and control mice at 1 day and 3 days after tMCAO (n =3 per group). **(D)** ICAM-1 expression in control and metformin-treated mice at 1 and 3 days after tMCAO. Arrows indicated representative ICAM-1 expression. Scale bar =100 μm. **(E)** p-NF-κB and NF-κB expression in saline- and metformin-treated groups at 1 day and 3 days after tMCAO. Bar graph show a quantification of p-NF-κB/NF-κB ratio (n =3 per group). Data are mean ± SD, *P < 0.05, metformin versus control, sham versus control group, **P < 0.01, metformin versus control group.

**Figure 6:**
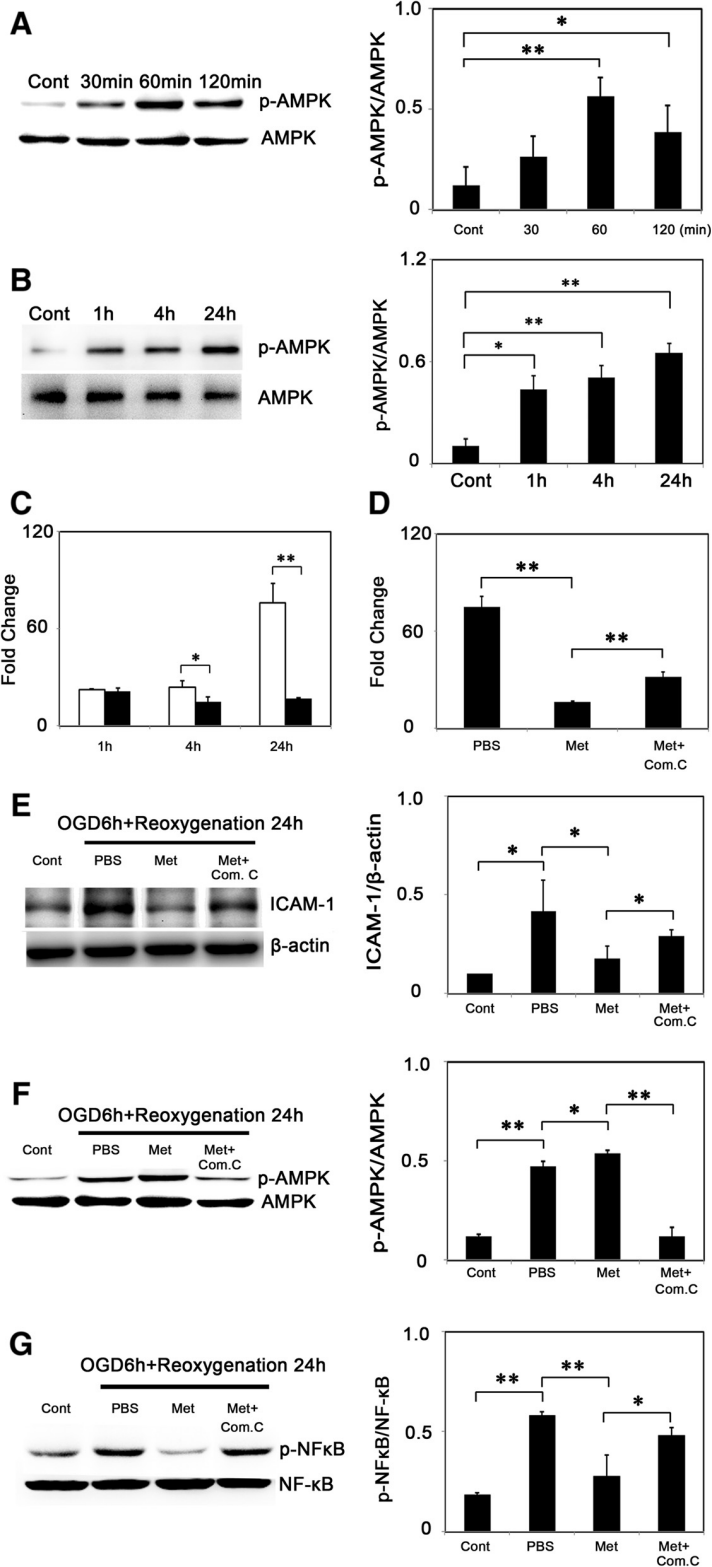
**Metformin promoted phosphorylation of AMPK and reduced ICAM-1 expression** ***in vitro*** **in an AMPK-dependent manner. (A)** p-AMPK and AMPK expression and quantification at 30, 60 and 120 minutes after metformin treatment *in vitro* in normal conditions. **(B)** p-AMPK and AMPK expression and quantification in control, 1, 4 and 24 hours in reoxygenation group in the presence of metformin *in vitro*. **(C)** Fold change of ICAM-1 expression in mRNA level 1, 4 and 24 hours after reoxygenation in saline- and metformin-treated group. **(D)** ICAM-1 expression in mRNA after metformin and AMPK inhibitor and compound C treatment in the oxygen glucose deprivation (OGD) model. **(E)** ICAM-1 expression and quantification after metformin, AMPK inhibitor and compound C treatment in OGD model. **(F)** p-AMPK and AMPK expression and quantification after metformin and compound C treatment in OGD model. **(G)** p-NF-κB and NF-κB expression and quantification after metformin and compound C treatment in OGD model. Data were mean ± SD, *P < 0.05, **P < 0.01. Representative results from three independent experiments are shown. Com. C = compound C.

To assess the phosphorylation status of AMPK at threonine residue, Western blot was used. We demonstrated that ischemia-reperfusion increased AMPK phosphorylation and this induction was increased after metformin treatment (Figure 5A). To further explore mechanisms of metformin in neuroprotection, we analyzed ICAM-1 expression after tMCAO. ICAM-1 was expressed constituently at low level in ECs, and after cerebral ischemia its expression was elevated hugely (Figure 5D). Furthermore, Western blot and RT-PCR results indicated that ICAM-1 was reduced after metformin-treatment compared to the control group (Figure 5B-C). We also found that metformin inhibited NF-κB phosphorylation at 1 day and 3 days after tMCAO (Figure 5E). To determine whether metformin-induced down-regulation of ICAM-1 was AMPK-dependent or not, we analyzed the effect of metformin at a cellular level, using OGD models to mimic *in vivo* ischemia/reperfusion injury. First, we treated bEND.3 cells with metformin to determine whether metformin could also increase p-AMPK *in vitro*. We found that there was an increase in p-AMPK expression at 60 and 120 minutes after metformin treatment: the maximum effect was at 60 minutes in normal conditions (Figure 6A) and while treatment with metformin after 6 hours of OGD, there was also an increase in p-AMPK expression 1 hour after reoxygenation and p-AMPK levels were unregulated for at least 24 hours after reoxygenation (Figure 6B). Second, we examined ICAM-1 expression after 6 hours of OGD and reoxygenation with or without metformin. After OGD/reoxygenation treatment, ICAM-1 expression was up-regulated in mRNA level and metformin inhibited this up-regulation: the inhibitive effects began at 4 hours after reoxygenation and were sustained for at least 24 hours after reoxygenation (Figure 6C). To test whether AMPK signaling was involved, a selective AMPK inhibitor, compound C, was used to block the AMPK phosphorylation. First, we found that compound C reduced metformin-induced AMPK phosphorylation, then, we used RT-PCR and Western blot to assess ICAM-1 expression after treatment. Results indicated that metformin reduced ICAM-1 expression in both mRNA and protein levels under OGD/reoxygenation conditions (Figure 6D-E). Furthermore, we used Western blot to evaluate the effects of metformin on NF-κB activation; the result indicated that metformin inhibited NF-κB phosphorylation and this function was abolished by compound C (Figure 6G). Thus, we concluded that metformin diminished ICAM-1 via an AMPK mediated signaling pathway and AMPK-NF-κB might be involved.

## Discussion

In the present study, we demonstrated that metformin protected the brain from ischemic injury through alleviating inflammatory responses in tMCAO mice, thus improving long-term recovery. Metformin diminished neutrophil infiltration, thereby alleviating endothelial injury and lowering BBB permeability. These effects were potentially mediated via an AMPK-dependent ICAM-1 down-regulation. We also found that inhibiting AMPK activation by compound C could reverse metformin-induced down-regulation of ICAM-1 *in vitro*. Thus, we concluded that metformin exerts its protective effect after cerebral ischemia partly through diminished ICAM-1 expression.

Metformin is a glucose-lowering agent, and is one of the first-line drugs recommended to treat type II diabetes mellitus [25]. The UK Prospective Diabetes Study (UKPDS) has revealed that metformin reduced the risk of all-cause mortality and stroke clinically; however, these benefits were independent of its anti-hyperglycemic effects, since metformin reduced glycated hemoglobin (HbA_1c_) to the same extent as sulphonylurea and insulin [5]. Metformin decreased myocardial injury in non-diabetic and diabetic mice [4] and prevented the progression of heart failure in dogs [10]. However, reports regarding neuroprotection of metformin in cerebral ischemia were controversial. Using a 90-minute tMCAO model, McCullough demonstrated that chronic treatment, both pre- and post- (3 weeks), with metformin reduced infarct volume effectively; however, pre-treatment (3 days) enhanced injury in ischemic stroke [26]. Harada *et al*. showed that using 3-day metformin treatment after 2 hours of tMCAO, metformin effectively reduced infarct volume [27], which was consistent with our result. In addition, Li has suggested that chronic treatment (14 days in drinking water, 300 mg/kg) with metformin in diabetic rats was protective, but acute treatment (1 day in drinking water, 300 mg/kg) exerted different effects [28]. However, we must note that different models were used. For acute treatment, 3-hour occlusion and 21 hours reperfusion model were used; however, for the chronic treatment, a 90-minute occlusion and 14 days reperfusion model was adopted, and glucose level was normal in chronic treatment rats. Thus, different animal strains and different models may explain the different effects observed from different studies [26]. In our study, using a 90-minute tMCAO model in mice and treatment at the time of reperfusion, we demonstrated that metformin could alleviate ischemic injury and improve neurobehavioral outcomes.

We demonstrated the protection by metformin on ischemic stroke involved, at least in part, AMPK activity [3]. However, McCullough’s group found that metformin enhanced ischemic injury in tMCAO animals in a 3-day precondition study through activating AMPK [26]. We believe this result can be due to the period of treatment. Metformin could increase AMPK phosphorylation both *in vitro* and *in vivo* [4,9]. We detected an induction of AMPK phosphorylation in both bEND.3 cells and the mouse brain after tMCAO. Notably, conditions that could activate AMPK have been proven to be beneficial in stress, particularly in ischemia. Ischemic precondition, which can activate AMPK due to an increase of the AMP/ATP *ratio*, is supposed to be protective in ischemic stroke [29]. Adiponectin reduces infarct size in cerebral ischemia and myocardial injury has been shown to be partly through promoting AMPK phosphorylation [30,31]. In addition, AMPK involves pleiotropic pathways that play critical roles in cerebral ischemia. Through suppression of the mTOR signaling pathway, AMPK regulates cell growth and autophagy [7]. Via activation of the Nrf2/SKN-1 signaling pathway, AMPK increases antioxidant gene expression [32]. By promotion of the eNOS pathway, AMPK reduces endothelial cell apoptosis and improves endothelial functions [9]. Furthermore, emerging evidence shows that AMPK is beneficial to neurons suffering from injuries such as ischemia, starvation, and oxidative damage [11]. Collectively, these results suggest that metformin-AMPK signaling pathways exert protective effects in stress conditions and protect the brain from ischemic stroke.

The mechanism by which metformin reduces inflammation after cerebral ischemia is poorly understood. ICAM-1 expression can be regulated by the nuclear transcriptional factor NF-κB [33]. Recently, the potency of metformin blocking NF-κB signaling has been illustrated [34]. In addition, metformin decreased TNF-α-induced ICAM-1 by inhibiting NF-κB activation in ECs [12]. Therefore, we suppose that metformin reduced ICAM-1 expression via the AMPK-NF-κB pathway. We also detected an inhibition of NF-κB activation after metformin treatment both *in vivo* and *in vitro*. Previous study showed that there was a huge increase of ICAM-1 expression at the time of reperfusion; in our study, metformin was administrated at the time of reperfusion. Increased expression of adhesion cytokine is detrimental during ischemic injury since it increases neutrophil adhesion to ECs and thus promotes their infiltration [16]. Anti-ICAM-1 treatment significantly reduced infarct volume [15]. Coincidently, we demonstrated that metformin decreased ICAM-1 both *in vivo* and *in vitro*, and this reduction in ICAM-1 *in vivo* was accompanied by alleviated neutrophil infiltration and reduced infarct size in tMCAO mice. Furthermore, our study indicated that this effect was possibly mediated by AMPK in a dependent manner. We concluded that metformin conferred resistance to ischemic stroke through decreasing ICAM-1 via the AMPK signaling pathway.

Besides increasing inflammation, neutrophil infiltration also induces EC injury and increases BBB permeability [35]. BBB disruption exacerbates brain injury after ischemia. Injuries such as ischemia and trauma lead to a disruption and reconstruction of ZO-1 and occludin, and an increase in BBB permeability [23]. Reduction of BBB permeability alleviates cerebral ischemia injury in both transient and permanent cerebral ischemia [19,22,23]. Recently, it has been reported that metformin-induced improvement of BBB functions in ECs *in vitro* is due to activating AMPK activity [24]. In the present study, we demonstrated that after metformin treatment, IgG and Evans blue leakage was significantly reduced and tight junction protein profoundly increased, leading to better outcomes in tMCAO mice.

## Conclusions

We demonstrated that metformin is beneficial to the treatment of ischemic stroke, which is possible through inhibiting inflammation via AMPK signaling pathways. Since metformin is a widely used drug with few adverse effects, using this long-established drug for a new use may be a promising way to develop an effective therapy for ischemic stroke [36]. Metformin has the potential to be useful in the clinical treatment of ischemic stroke.

## Additional files

Additional file 1: Table S1. Metformin did not influence blood gas, glucose level and body weight 14 days after transient middle cerebral artery occlusion (tMCAO) in mice. Additional file 2: Figure S1. Metformin reduced neutrophil infiltration in transient middle cerebral artery occlusion (tMCAO) mice. Gr1 (Ly 6G)^+^ cells (arrows) and their quantification in control and metformin treated mice at 1 and 3 days following tMCAO (n = 3/group). Scale bar = 100 μm. Data are mean ± SD, ***P* <0.01, metformin versus control group.

## Abbreviations

AMPK: activating AMP-activated kinase
BBB: blood-brain barrier
DAB: 3,3’-diaminobenzidine
ECA: external carotid artery
ECs: endothelial cells
ELISA: Enzyme Linked Immunosorbent Assay
FBG: fasting blood-glucose
ICA: internal carotid artery
ICAM-1: intercellular adhesion molecule-1
IOD: integrated optical density
MCA: middle cerebral artery
mNSS: modified Neurological Severity Scores
MPO: myeloperoxidase
OGD: oxygen-glucose deprivation
tMCAO: transient middle cerebral artery occlusion
VCAM-1: vascular cell adhesion molecule-1
