# Supplementary material for: Metformin attenuates blood-brain barrier disruption in mice following middle cerebral artery occlusion
Source: J Neuroinflammation. 2014 Oct 15;11:177. doi: 10.1186/s12974-014-0177-4 (PMC4201919; doi:10.1186/s12974-014-0177-4)
Supplement: Additional file 1: Table S1. — Metformin did not influence blood gas, glucose level and body weight 14 days after transient middle cerebral artery occlusion (tMCAO) in mice. [file 12974_2014_177_MOESM1_ESM.docx]

**Metformin attenuates blood-brain barrier disruption in mice following middle cerebral artery occlusion**

Yanqun Liu^1^, MS; Guanghui Tang^2^, MS; Yaning Li^2^, MS; Yang Wang^1^, MS; Xiaoyan Chen^2^, MS; Xiang Gu^2^, MS, Zhijun Zhang^2^, PhD; Yongting Wang^2^, PhD; and Guo-Yuan Yang^1,2^, MD, PhD

**Additional file: Table S1: Metformin did not influence blood gas, glucose level and body weight 14 days after tMCAO in mice.**


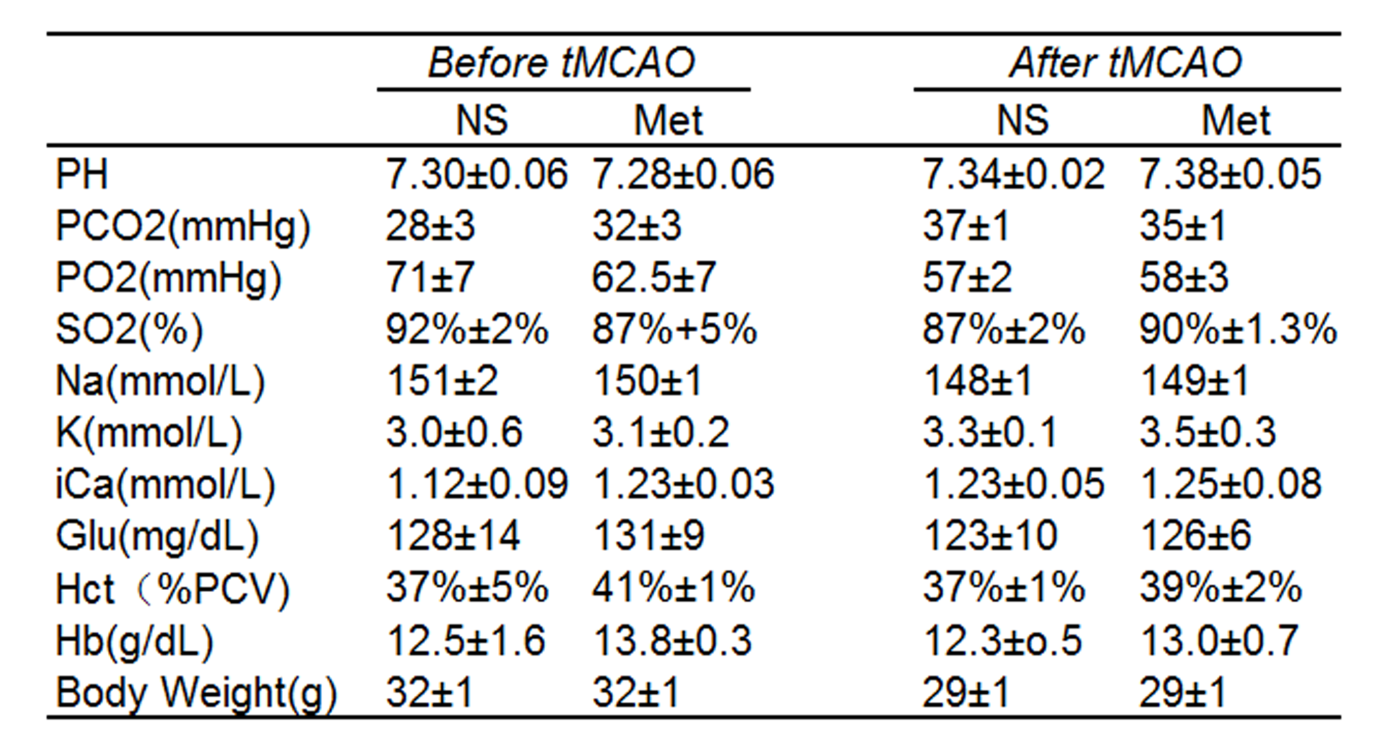


Table showed vein blood gas analysis results, glucose levels and body weight at 1 day before tMCAO and at 14 days after tMCAO in metformin and saline treated mice (n = 4 per group). Data were mean ± SD.
