# Supplementary figures and images for: Metformin attenuates blood-brain barrier disruption in mice following middle cerebral artery occlusion
Source: J Neuroinflammation. 2014 Oct 15;11:177. doi: 10.1186/s12974-014-0177-4 (PMC4201919; doi:10.1186/s12974-014-0177-4)

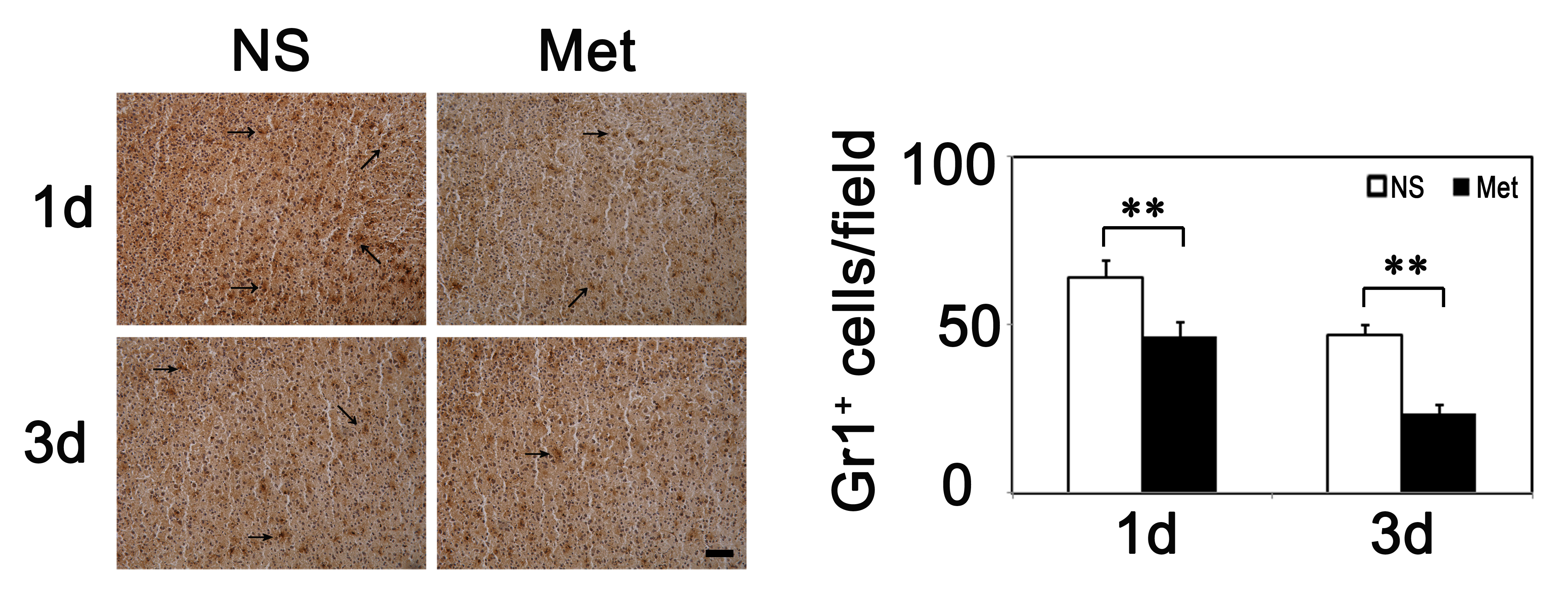

Supplement: Additional file 2: Figure S1. — Metformin reduced neutrophil infiltration in transient middle cerebral artery occlusion (tMCAO) mice. Gr1 (Ly 6G)+ cells (arrows) and their quantification in control and metformin treated mice at 1 and 3 days following tMCAO (n = 3/group). Scale bar = 100 μm. Data are mean ± SD, **P <0.01, metformin versus control group. [file 12974_2014_177_MOESM2_ESM.tiff]
